# Quality control of multiplex antibody detection in samples from large-scale surveys: the example of malaria in Haiti

**DOI:** 10.1038/s41598-020-57876-0

**Published:** 2020-01-24

**Authors:** Lotus L. van den Hoogen, Jacquelin Présumé, Ithamare Romilus, Gina Mondélus, Tamara Elismé, Nuno Sepúlveda, Gillian Stresman, Thomas Druetz, Ruth A. Ashton, Vena Joseph, Thomas P. Eisele, Karen E. S. Hamre, Michelle A. Chang, Jean F. Lemoine, Kevin K. A. Tetteh, Jacques Boncy, Alexandre Existe, Chris Drakeley, Eric Rogier

**Affiliations:** 1 Department of Infection Biology, London School of Hygiene & Tropical Medicine, London, UK; 2 Laboratoire National de Santé Publique, Port-au-Prince, Haiti; 3 Centre of Statistics and Applications, University of Lisbon, Lisbon, Portugal; 4 Center for Applied Malaria Research and Evaluation, Tulane University School of Public Health & Tropical Medicine, New Orleans, Louisiana USA; 5 Department of Social and Preventive Medicine, University of Montreal School of Public Health, Montreal, Canada; 6 Malaria Branch, Division of Parasitic Diseases and Malaria, Centers for Disease Control and Prevention, Atlanta, Georgia USA; 7 CDC Foundation, Atlanta, Georgia USA; 8 Ministère de la santé publique et de la population, Port-au-Prince, Haiti

**Keywords:** Malaria, Malaria, Parasite host response, Infectious-disease diagnostics

## Abstract

Measuring antimalarial antibodies can estimate transmission in a population. To compare outputs, standardized laboratory testing is required. Here we describe the in-country establishment and quality control (QC) of a multiplex bead assay (MBA) for three sero-surveys in Haiti. Total IgG data against 21 antigens were collected for 32,758 participants. Titration curves of hyperimmune sera were included on assay plates, assay signals underwent 5-parameter regression, and inspection of the median and interquartile range (IQR) for the y-inflection point was used to determine assay precision. The medians and IQRs were similar for Surveys 1 and 2 for most antigens, while the IQRs increased for some antigens in Survey 3. Levey-Jennings charts for selected antigens provided a pass/fail criterion for each assay plate and, of 387 assay plates, 13 (3.4%) were repeated. Individual samples failed if IgG binding to the generic glutathione-*S*-transferase protein was observed, with 659 (2.0%) samples failing. An additional 455 (1.4%) observations failed due to low bead numbers (<20/analyte). The final dataset included 609,438 anti-malaria IgG data points from 32,099 participants; 96.6% of all potential data points if no QC failures had occurred. The MBA can be deployed with high-throughput data collection and low inter-plate variability while ensuring data quality.

## Introduction

Measurement of antibody responses to malaria at the population-level can describe recent and historical transmission patterns^1–4^ and is informative for malaria research and program policies^5–7^. Antibodies can be quantitatively measured by a variety of techniques including the enzyme-linked immunosorbent assay (ELISA) and multiplex bead assays (MBAs). The latter allows the simultaneous detection of antibodies to multiple antigenic targets and has been utilized now for *Plasmodium* serology for over a decade^8^. Since the advent of *Plasmodium-*specific MBAs, numerous assay optimisation and implementation studies^9–17^, as well as epidemiological application studies^18–21^, have been published by various groups.

In generating responses to many antigens simultaneously, MBAs have the advantage of reducing needed reagent quantities, sample volume, and time in the laboratory compared to the ELISA^8,9^. Assays of any type require controls or standards to assess variability across runs or batches and to compare research studies, and with a broad panel of antigens in the MBA, it is potentially difficult to find standards for all targets being assayed. Recently, standardization using curves of known concentrations of total human IgG has been suggested, but this was problematic in showing insufficient reproducibility between operators^13^. Moreover, these do not allow for the assessment of antigen-specific responses which are important for quality control in assessing the stability of specific antibodies over time. In-house pools of hyperimmune sera are commonly employed for serological studies, and recently, a World Health Organization (WHO) reference *Plasmodium falciparum* (*Pf*) serological standard has been developed by the National Institute for Biological Standards and Control (NIBSC)^22^. This standard has previously been tested in a MBA panel of 40 malarial and non-malarial antigens^15^.

Previous studies on the application and validation of the MBA have shown its correlation with ELISA^8,10,12,17,23^, stability and reproducibility of coupled beads^10,12,16,24^, use of detection antibodies for Ig classes^15^ and IgG subclasses^11,15^, as well as consistency between mono- versus multiplex results^10,12,15–17^. Although intra- and inter-assay variability for antimalarial antibodies have been discussed before^10,13,14,16,25^, and analytical methodologies have been evaluated to determine inter-assay variability^24,26,27^, few have formally assessed variability in large-scale population surveys (i.e., studies involving thousands to tens of thousands of samples over time)^16^. Here, we discuss the in-country establishment and quality control process for multiplex antimalarial antibody (IgG) detection for multiple large-scale malaria surveys performed in Haiti in 2017.

## Results

### Assay throughput

Dried blood spots (DBS) were collected in three cross-sectional surveys in central and southwestern Haiti in 2017^28^. IgG antibody responses were successfully collected across 21 antigens (Table 1; 17 *P. falciparum* antigens, 2 non-*P. falciparum Plasmodium* antigens and 2 non-malarial antigens) using a multiplex bead assay (MBA; see methods and^29^). From all collected survey samples that were processed at the Haitian national laboratory, minor loss of field samples was found due to data management issues (e.g., incorrect barcodes due to accidental typing while scanning barcodes in the laboratory or no blood sample recorded/collected in the field) or loss of DBS between field collection and laboratory assessment (Table 2). Thus, the majority of samples provided data appropriate for analyses: 99.2% (5,956/6,006) for Survey 1; 99.6% (21,801/21,891) for Survey 2; and 99.3% (5,001/5,034) for Survey 3. Laboratory work involved 71 assay plates over five weeks for Survey 1; 257 plates over nine weeks for Survey 2; and 59 plates over four weeks for Survey 3. Together these represent 32,758 participant samples processed over an eighteen-week period. After removal of median fluorescence intensity (MFI) data across all analytes for samples with missing or high responses to the generic glutathione-*S*-transferase (GST, n = 659, 2.0%; Supplementary Fig. 1), 5,898 samples passed QC in Survey 1 (99.0% of those received at the laboratory); 21,234 samples in Survey 2 (97.4%); and 4,967 samples in Survey 3 (99.3%). Removal of single analytes’ datapoints due to low bead counts accounted for additional minor loss of data (n = 455 observations, 0.07% of all observations). Following these QC checks, there were 673,624 unique IgG observations across the 21 included antigens (Table 2).

**Table 1:** Characteristics of multiplex bead assay antigen panel for three malaria transmission surveys in Haiti.

Antigen	Alias	Pathogen	Description	Location	Purification Tag	Strain	Rationale	Coupling Conc. (µg/mL beads)	Coupling pH	Reference
Etramp 4 Ag 2	etr42	*P. falciparum*	Early transcribed membrane antigen	iRBC, PVM	GST	3D7	Recent *Pf* exposure*	115	7.2	^4^; K.K.A. Tetteh unpublished
Etramp 5 Ag 1	etr51	*P. falciparum*	Early transcribed membrane antigen	iRBC, PVM	GST	3D7	Recent *Pf* exposure*	100	7.2	^35^; K.K.A. Tetteh unpublished
GEXP18	gexp	*P. falciparum*	Gametocyte exported protein 18	iRBC/Gametocyte	GST	3D7	Recent *Pf* exposure*	200	7.2	^4^; K.K.A. Tetteh unpublished
H103	h103	*P. falciparum*	H103/merozoite surface protein 11	Merozoite surface/rophtry neck	GST	3D7	*Pf* exposure	100	7.2	^36^
HRP2	hrp2	*P. falciparum*	Histidine rich protein 2	iRBC and secreted	GST	Type A and B	*Pf* exposure	25	5.0	^37^
HSP40 Ag1	hsp40	*P. falciparum*	Heat shock protein 40	iRBC	GST	3D8	Recent *Pf* exposure*	100	7.2	^4^; K.K.A. Tetteh unpublished
Hyp 2	hyp2	*P. falciparum*	Plasmodium exported protein	Hypothesised location: iRBC	GST	3D7	Recent *Pf* exposure*	1000	7.2	^4^; K.K.A. Tetteh unpublished
LSA-1	lsa1	*P. falciparum*	Liver surface antigen 1	Infected hepatocyte	N/A	Synthesized peptide, Pl1043 epitope	*Pf* exposure (liver stage)	60	5.0	^38^
MSP2 CH150/9	msp2_ch150	*P. falciparum*	CH150/9 allele of MSP2; full-length	Merozoite surface	GST	CH150/9	*Pf* exposure	5	5.0	^39^
MSP2 Dd2	msp2_dd2	*P. falciparum*	Dd2 allele of MSP2; full-length	Merozoite surface	GST	Dd2	*Pf* exposure	20	5.0	^40^
PfAMA1	ama1	*P. falciparum*	Apical membrane antigen 1	Micronemes	His	FVO	*Pf* exposure	15	7.2	^41^
PfGLURP R0	glurp0	*P. falciparum*	Glutamate rich protein R0	Merozoite surface	N/A	Synthesized peptide, R0 fragment	*Pf* exposure	30	5	^18^
PfGLURP R2	glurp2	*P. falciparum*	Glutamate rich protein R2	Merozoite surface	His_x6_	F32	*Pf* exposure	15	7.2	^42^
PfMSP-1_19_	msp119	*P. falciparum*	19 kDa fragment of MSP1 molecule	Merozoite surface	GST	Wellcome	*Pf* exposure	20	7.2	^43^
PfSEA1	sea	*P. falciparum*	Schizont egress antigen	iRBC	GST	3D7	*Pf* exposure	20	5	^44^; K.K.A. Tetteh unpublished
PmMSP-1_19_	pmmsp119	*P. malariae*	19 kDa fragment of MSP1 molecule	Merozoite surface	GST	Pm China I	*Pm* exposure	20	5	^45^
PvMSP-1_19_	pvmsp119	*P. vivax*	19 kDa fragment of MSP1 molecule	Merozoite surface	GST	Pv Belem	*Pv* exposure	20	5	^45^
rCSP	rcsp	*P. falciparum*	Circumsporozoite surface protein	Sporozoite	N/A	3D7	Recent *Pf* exposure* (sporozoite stage)	60	7.2	^46^
SBP1	sbp1	*P. falciparum*	Skeleton-binding protein; Maurer’s cleft.	iRBC	GST	3D7	*Pf* exposure	15	5	^47^; K.K.A. Tetteh unpublished
GST	gst	*S. japonicum*	Glutathione S-transferase				Correct for background reactivity due to GST-tag	20	5	J. Priest/CDC
Tetanus Toxoid	tt	*C. tetani*	Tetanus Toxoid				Vaccination target: internal “positive” control	12.5	5	Massachusetts Biologic Laboratories

*Associated with recent *Pf* exposure as described in ref. ^4^. Conc.: concentration. iRBC: infected red blood cell. PVM: parasitophorous vacuole membrane. kDa: k ilodalton. *Pf*: *Plasmodium falciparum*. *Pv*: *Plasmodium vivax*. *Pm*: *Plasmodium malariae*. N/A: not applicable.

**Table 2:** Number of samples and observations for which Immunoglobulin G (IgG) antibody responses were successfully collected using a multiplex bead assay across three malaria transmission surveys in Haiti.

	Survey 1	Survey 2	Survey 3
**Plates, n**	71	257	59
**Samples, n** Proportion of previous n			
Collected in the field	6006	21891	5034
Received/processed at the lab	5956 99.17%	21801 99.59%	5001 99.34%
GST reading available	5922 99.43%	21336 97.87%	4989 99.76%
Acceptable GST reactivity	5898 99.59%	21234 99.52%	4967 99.56%
Observations*, n Loss, n			
All antigens (n = 21) Loss	123,850 8	445,787 127	103,987 320
*Plasmodium* antigens (n = 19) Loss	112,054 8	403,325 121	94,059 314
*P. falciparum* antigens (n = 17) Loss	100,260 6	360,872 106	84,137 302

*Unique IgG observations successfully collected (i.e. number of participants multiplied by number of antigens/peptides to which antibody responses were collected). GST: glutathione-*S*-transferase.

### Robust responses for both positive control standards

**Figure 1:**
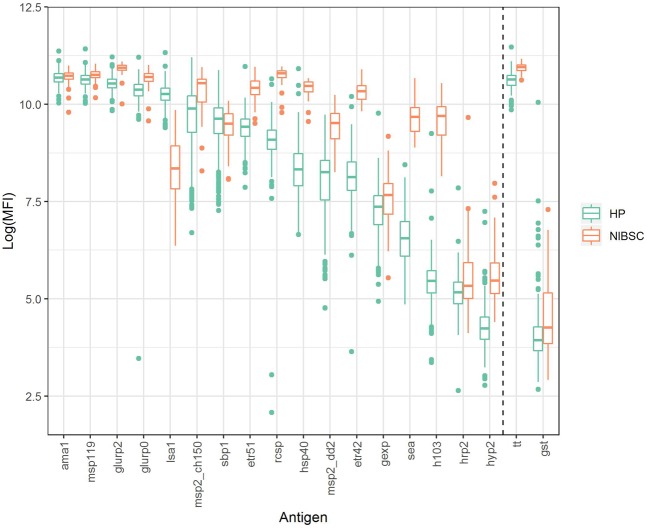
Antibody reactivity profile of hyperimmune sera standards used in this study. MFI: Median fluorescence intensity; values were corrected for background reactivity of blank responses and natural log transformed (y-axis). HP: Haitian hyperimmune sera pool (for details see main text). NIBSC: WHO *Plasmodium falciparum* 10/198 NIBSC standard. The HP curve was run on every plate, while the NIBSC curve was run on one plate per day. Responses to the first point of the curve are shown, with a serum concentration of 1:200 for the HP and 1:100 for the 10/198 standard. For antigen (x-axis) abbreviations see Table 1, antigens are ordered by descending median HP responses. In addition to malarial antigens, tetanus toxoid (tt) and glutathione S-transferase (gst) responses are shown (right side of dashed vertical black line).

A Haitian positive serum control pool (HP) was created using country-wide DBS samples from individuals with confirmed malaria (n = 63) previously collected during healthcare visits. A 6-point, 5-fold titration curve of the HP standard starting at 1:200 was included on every plate, while one using the WHO *Pf* 10/198 NIBSC standard^22^ was included on one plate per day (starting at 1:100). The highest concentrations of both the HP and the NIBSC positive control standard curves showed robust IgG responses for nearly all of the included *Pf* antigens (Fig. 1). Generally higher MFI responses were seen in the NIBSC standard, likely due in part to the higher serum concentration. The lowest MFI responses were recorded to the HRP2 and Hyp2 antigens in both standards (median MFI < 500 before log-transformation).

### Inter-plate variability

**Figure 2:**
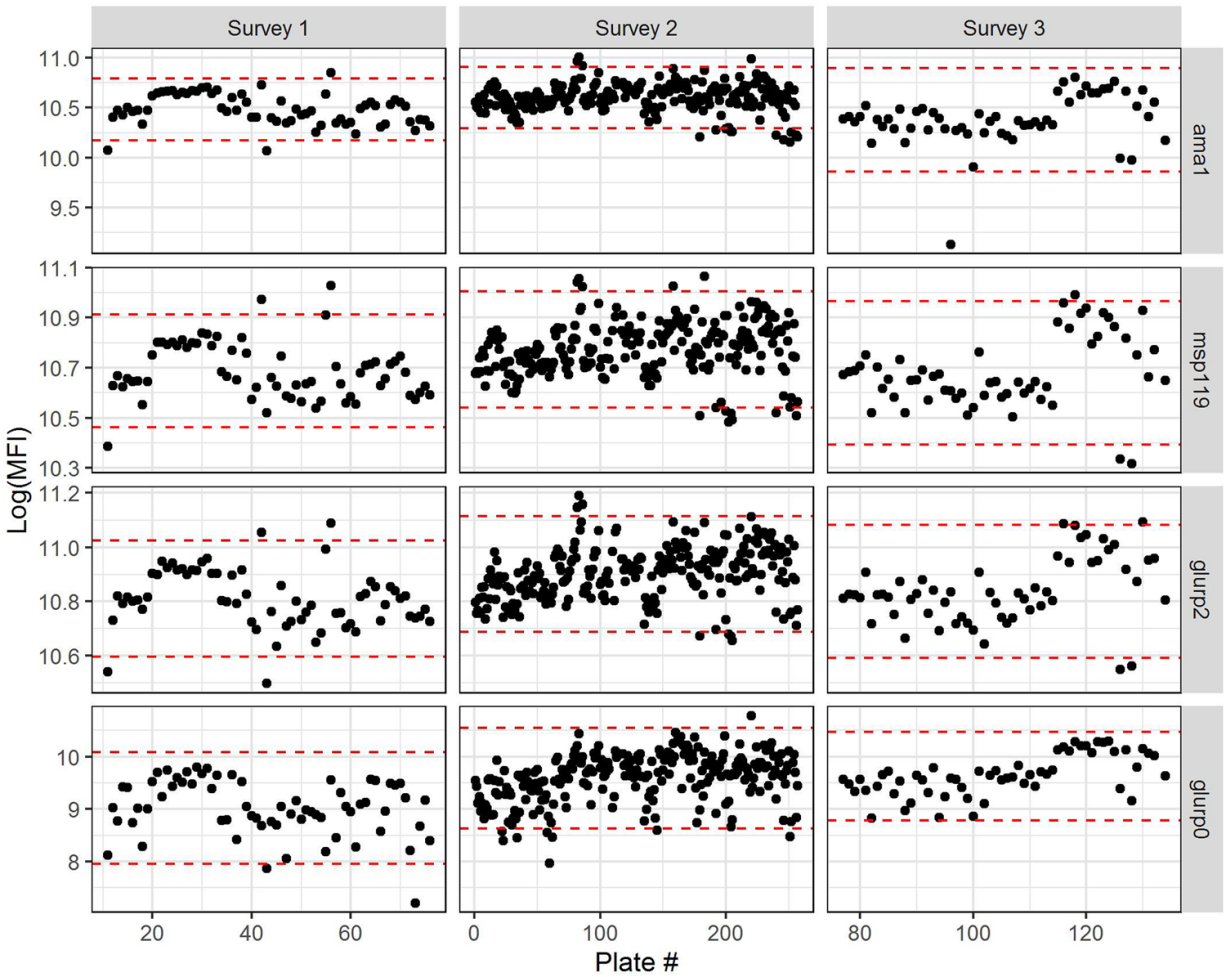
Levey-Jennings charts of antibody responses in the standard of Haitian hyperimmune sera pool across all plates per survey. MFI: Median fluorescence intensity; values were corrected for background reactivity of blank responses and natural log transformed. HP: Haitian hyperimmune sera pool (for details see main text). Responses in the third dilution point of the curve (serum concentration of 1:5,000) per plate are shown across three surveys. The mean plus/minus two times the standard deviation of responses in the third dilution point of the curve per survey and antigen are shown in dashed red lines.

**Figure 3:**
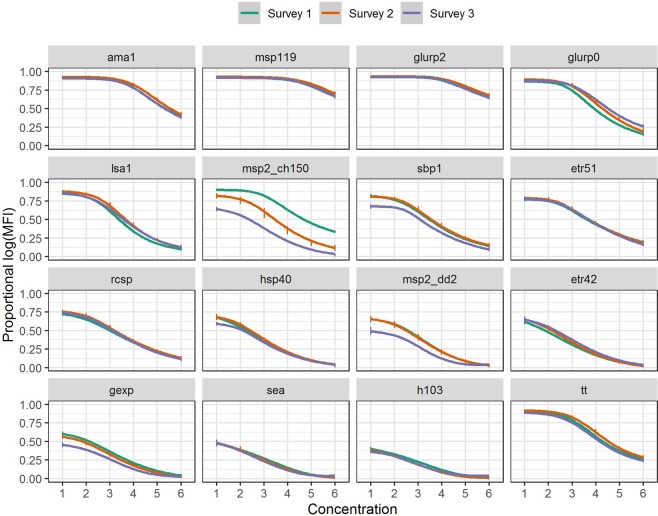
Average standard curves of the standard of Haitian hyperimmune sera pool for each survey. MFI: Median fluorescence intensity; values were corrected for background reactivity of blank responses and natural log transformed. HP: Haitian hyperimmune sera pool (for details see main text). MFI values were converted to proportions using the minimum and maximum MFI value for all standard curves across all antigens (2.07 and 11.17 respectively). For each plate and antigen, standard curves were fitted using 5-parameter logistic regression. Standard curves were only fitted if the non-log-transformed MFI of at least one of the dilution points was larger than 100. Using the curve parameters, MFI values were predicted across a sequence of 200 values of standard curve concentrations for each of the plates. Standard curves per survey were plotted using the generalized additive model method and the interquartile range is shown in vertical lines at each of the dilution steps of the standard curve. For antigen abbreviations see Table 1, malaria antigens are ordered from top left to bottom right by median responses as shown in Fig. 1. In addition to malarial antigens, results for tetanus toxoid (tt) are shown.

**Figure 4:**
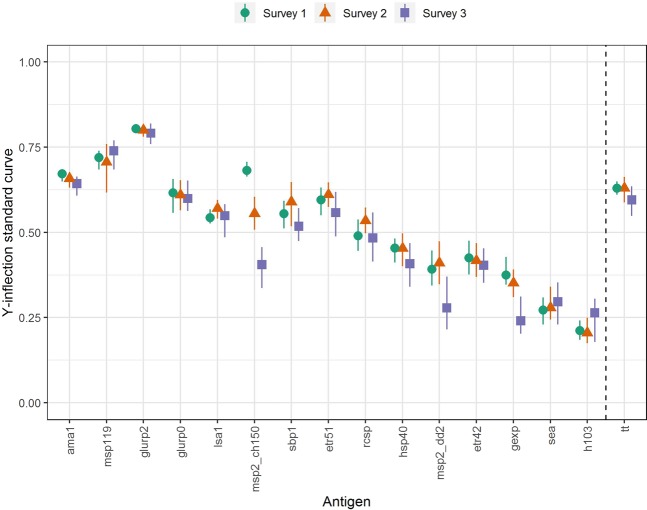
Median and interquartile range of predicted y-inflection points of standard curves per survey using the standard of the Haitian hyperimmune sera pool. Median (shapes) and interquartile range (vertical lines) of the predicted y-inflection points from standard curves across all plates using 5-parameter logistic regression are shown per survey. For antigen (x-axis) acronyms see Table 1, malaria antigens are ordered by median responses as shown in Fig. 1. In addition to malarial antigens, results for tetanus toxoid (tt) are shown (right side of dashed vertical black line).

Levey-Jennings plots of IgG responses of the third point of the HP standard curve are shown in Fig. 2. Values on assay plates that fell outside of the 2 standard deviation (SD) range of mean responses for two out of three highly immunogenic antigens (GLURP-R2, PfAMA-1 and PfMSP-1_19_) were selected to be repeated: 2 plates in Survey 1, 9 plates in Survey 2, and 2 plates in Survey 3 (13/387 total assay plates, 3.4%). Upon repetition, all 13 of these assay plates passed the QC check and provided useable serological data. When comparing 2 to 5 parameter logistic regression fits for standard curves values, the 5-parameter logistic regression fit showed the smallest sum of squared errors for the HP curve for the majority of antigens and plates (≥88%; Supplementary Table 1) and thus was used for all standard curves for further analysis. HP standard curves per survey are shown in Fig. 3 for all antigens except HRP2 and Hyp2 (which were unable to be fitted to curves), and curves for the NIBSC standard are shown in Supplementary Fig. 2. Inspection of the median and IQR of the y-inflection point was used to assess within and between survey variation in standard curves (Fig. 4). The median and length of the IQR of y-inflection points was similar for Survey 1 and Survey 2 for most antigens; except for a smaller recorded Survey 2 median for MSP2_CH150/9 (i.e., below the 25^th^ percentile of Survey 1) as well as a larger Survey 2 IQR for PfMSP-1_19_, MSP2_CH150/9, SBP1 and to a lesser extent MSP2_Dd2. The length of the IQR for y-inflection points was generally highest in Survey 3. While for most antigens the median Survey 3 y-inflection point was similar to Survey 1 and 2, a smaller Survey 3 median (i.e., below the 25^th^ percentile of Survey 2) was recorded for MSP2_CH150/9, MSP2_Dd2, GEXP18 and borderline for SBP1. Standard curves for *Pf*, *P. vivax* (*Pv*) and *P. malariae* (*Pm*) MSP-1_19_ antigens with the HP and NIBSC standard are shown in Supplementary Fig. 3.

## Discussion

In this study set in Haiti, we described the in-country establishment and quality control of an MBA simultaneously detecting IgG responses to 17 *P. falciparum* recombinant antigens and peptides and 4 additional non-*P. falciparum* antigens. Laboratory antibody measurements were collected for 32,758 participant samples across three surveys in eighteen weeks. Results for only 0.2–0.5% of the participants for each of the three surveys had to be removed due to high GST responses (i.e., evidence for non-specific IgG binding). The data collected for the remaining participants represent 545,683 *P. falciparum* serological data points of which only 414 (0.08%) had to be removed due to well-specific errors such as low bead counts. The quality control to assess the inter-plate precision of the assay was based on a specifically created positive control standard of RDT positive Haitians with high titers of anti-*Pf* antibodies (Haitian hyperimmune sera pool: HP). The rationale for creation of the HP standard was that it would most accurately represent seroresponses of the Haitian populace to the locally circulating *P. falciparum* parasites. In addition, we included the WHO *Pf* 10/198 NIBSC reference standard on one plate per day to compare for consistency among days^22^. The MBA was implemented in this setting as a high-throughput tool enabling rapid turnaround of antibody measurements for malaria epidemiological surveys.

Inter-plate variability was assessed using Levey-Jennings plots which showed no trends in loss or gain of IgG responses in the HP standard over time. Minor plate-to-plate variation in MFI values was observed during assay processing, and could have been caused by inter-technician variability, pipetting errors or fluctuations in laboratory temperatures and/or incubation time during assay processing. For this specific study, plate values that fell outside the mean +/−2 SD of responses in the third point of the HP standard curve (i.e., the third point in the six-point dilution series of the HP standard) for two out of three highly immunogenic targets were repeated as previously described by others^16^. Using multiple targets for this selection compared to one target avoids rejecting a plate due to well-specific errors such as low bead counts or pipetting errors. This multi-antigen methodology also allows for pragmatic selection of truly problematic assay plates, since comprehensive errors (i.e., adding wrong amounts of detection reagents, improper incubation time) would affect all antigens’ MFI readings on the entire plate and decreased or elevated signals would be seen throughout the whole antigen panel.

Inter-plate variability was further assessed using 5-parameter logistic regression for standard curves on each plate^26,30^. Inspection of the median and IQR of the y-inflection point was used to assess within and between survey variation in standard curves. The median y-inflection points were similar for Survey 1 and Survey 2 for most antigens with narrow IQRs, and the length of the IQR for y-inflection points was generally highest in Survey 3. For four of the included targets, the standard curves revealed a loss of reactivity over time (MSP2_Dd2, MSP2_CH150/9, GEXP18 and SBP1). As beads were coupled in one batch at the start of Survey 1 to exclude variations between bead batches, the loss in reactivity may be explained by these antigens covalently bound to the microbeads as being less stable after long-term storage, or degradation or loss of binding capacity of the IgG antibodies in the HP standard. Therefore, comparison of results among the three surveys for these targets should be interpreted with caution and future use of these antigens would need to optimise storage and binding conditions.

The application of the WHO *Pf* 10/198 NIBSC standard to the MBA was recently described by Ubillos *et al*. where they showed robust IgG responses to 23 antigens, 20 of which were previously described malarial antigenic targets^15^. Here, we reported IgG antibody responses in this reference standard to 14 novel recombinant malarial antigens of which 12 showed robust responses (IgG against Hyp2 and HRP2 did not). Antibody responses to Pf-, Pv- and Pm-MSP-1_19_ antigens were similar to those described when the reference standard was developed and tested on ELISA by showing high titers for the Pf- and Pm-MSP-1_19_ targets, and negligible IgG against PvMSP-1_19_^22^. By adding this WHO *Pf* 10/198 NIBSC standard to one plate per day alongside the newly developed hyperimmune pool of Haitian sera on every plate, we were able to confirm the presence or absence of trends over time in antigen-specific results. However, standard curves from this NIBSC standard pool were more variable between plates and surveys. This could partly be explained by the smaller sample size (i.e., less curves were run for the entire study), stability of the sera over time, or potentially this standard may be more sensitive to day-to-day variations in incubation times during assay processing.

Others have shown that combining antibody responses to multiple targets more accurately reflects recent malaria infection than to one antigen though at small increments^4,31,32^. The fact that this study showed that multiplex antimalarial antibody data could be collected accurately at scale aids in ensuring representation of the variation in human immune responses. Additional longitudinal studies collecting multiplex antimalarial antibody data following natural infections across varied settings are needed to identify which antigens best reflect exposure histories.

In this study, we have described the successful in-country establishment of the MBA with highly efficient throughput and acceptable inter-plate variability for well-characterised malaria antigenic targets in Haiti. This assay allows for rapid assessment of the exposure history of populations which can directly inform malaria stratification and targeting of interventions. However, inter-plate variability was considerable for some of the newly described targets with lower immunogenicity. IgG antibodies to these targets are perhaps more sensitive to long-term storage, fluctuations in laboratory temperatures and/or incubation time during assay processing. Future work should focus on further optimisation of international assay standards and standardized quality assurance/quality control metrics for multiplex antibody detection assays.

## Methods

### Study population

Three cross-sectional surveys were conducted in 2017 in Haiti: two in the Artibonite valley of central Haiti (Survey 1 in May-Jun and Survey 2 in Jul-Oct with a two-week pause due to hurricanes), and one in Grand’Anse department in south-western Haiti (Survey 3 in Nov-Dec). In 2017, malaria incidence in the Grand’Anse department was estimated at 18.1 per 1,000 inhabitants while this was 0.6 per 1,000 in Artibonite (source: National Malaria Control Program, PNCM, Haiti). Survey design and enrollment procedures have been described elsewhere^28^. The number of participants providing a blood sample in each survey included, Survey 1: 6,006 participants, Survey 2: 21,891 and Survey 3: 5,034. In the former two surveys, finger-prick capillary blood was collected in microtubes (Safe-T-Fill Capillary Blood Collection Systems: EDTA, RAM Scientific Inc.) and pipetted on Whatman 903 cards (GE Healthcare) within 24 hours, whereas in the latter survey, blood was spotted directly onto the Whatman 903 cards at point-of-contact. In all surveys, cards were dried overnight and packed the next day in individual bags with silica gel. These dried blood spots (DBS) were kept at room temperature and were transported to the national laboratory (*Laboratoire National de Santé Publique*, LNSP) in Port-au-Prince once per week where they were stored at 4 °C until processed. Participants were also tested with a rapid diagnostic test (RDT, SD Bioline Malaria Antigen Pf.; 05FK50) and treated according to national guidelines if positive^28^.

### Antigen coupling to beads

Antigens were covalently coupled to unique bead regions as previously described by Rogier *et al*.^19,29^. In addition to the malarial antigen panel, the glutathione-*S*-transferase (GST) protein was included as a generic antigen to correct for potential non-specific binding. In addition, this served as an internal control as many of the recombinant malaria antigens were GST fused. Tetanus toxoid (TT, Massachusetts Biologic Laboratories) was included to act as an internal positive control as vaccinated Haitians would show responses to this target. Antigen characteristics and details on antigen to bead coupling conditions are depicted in Table 1.

### Assay standards

A Haitian positive serum control pool (HP) was created using country-wide DBS samples from RDT positive individuals collected during healthcare visits. Blood spots from 63 participants with high responses to a range of *Pf* antigens were combined and eluted in Buffer B (phosphate-buffered saline (PBS) containing 0.5% BSA, 0.05% Tween 20, 0.02% sodium azide, 0.5% polyvinyl alcohol, 0.1% casein, 0.8% polyvinylpyrrolidone and 0.5% w/v *E. coli* extract) to a whole blood dilution of 1:50 which corresponds to serum dilution of approximately 1:100. The approximate serum concentration is used to indicate the dilution factor throughout the remainder of this report. A 6-point titration curve of the Haitian hyperimmune sera was created in bulk, stored at 4 °C and used on each assay plate. The first point of this HP curve was a dilution of 1:100 and titrated by 5-fold, meaning the second point was 1:500 up to the sixth point at 312,500. The WHO *Pf* 10/198 NIBSC standard^22^ was eluted in 1.0 ml of dH20 (1:5 serum concentration, 100 units) and diluted further in Buffer B. As with the HP curve, a 6-point curve (starting at 1:50) of 5-fold dilutions was prepared in bulk for use throughout the whole study and stored at 4 °C. As samples and controls were diluted 1:2 into assay plates (see below), final starting concentrations for the first points of the titration curves were 1:200 (HP) and 1:100 (NIBSC) and remaining dilution points in titration curves followed accordingly. Unlike the HP curve, the NIBSC standard curve was only run on a single assay plate every day. In addition, two blanks (buffer B only) were run on each plate.

### Multiplex bead assay

Sample preparation and the MBA for data collection have been described elsewhere^29^. Briefly, blood elution for each sample was completed by taking one 3 mm spot from the centre of a DBS and eluting overnight in 173 µl of buffer B to create a 1:100 serum dilution for each sample. Samples were sealed in holding plates for storage at 4 °C and tested within three weeks of blood elution. For the assay, a mixture of all bead regions was prepared by adding 6 µl per coupled bead region (62,500 beads/antigen/plate) in 5 ml of Buffer A (PBS containing 0.5% BSA, 0.05% Tween-20, 0.02% sodium azide) for each plate. The bead mixture was mixed using a serological pipette and 50 µl was added to each well of a 96-well BioPlex Pro plate (Bio-Rad). Plates were placed on handheld magnetic separators (Luminex Corporation) and washed two times with wash buffer (PBS containing 0.05% Tween-20). After removing plates from the separator, 50 µl of reagent mixture in Buffer A (1:500 biotinylated anti-human IgG, Southern Biotech; 1:625 biotinylated anti-human IgG_4_, Southern Biotech; and 1:200 Streptavidin conjugated to phycoerythrin, Invitrogen) was added to each well followed by 50 µl of eluted blood sample. Plates were incubated on a shaker overnight at room temperature at 600 rpm protected from light. The next day, plates were washed three times and 100 µl PBS was added to resuspend beads. Plates were shaken lightly for 30 minutes and read with the MAGPIX machine (Millipore) using Bio-Plex Manager MP (Bio-Rad) software with a target of 50 beads/antigen/well. Median fluorescence intensity (MFI) was recorded for each sample and corrected for background reactivity by subtracting blank values on each plate (Buffer B only) by antigen (MFI corrected for background, hereafter: MFI). Results were exported to Excel workbooks by plate.

### Statistical analyses

All statistical analyses were performed in R Studio version 3.3.3^33^. Participant samples with GST MFI levels above a threshold of 1,000 were excluded from further analyses as evidence of non-specific binding. All MFI values were log transformed with MFI values smaller than background responses replaced with the median background values for all antigens (background non-log transformed MFI of 7.90; interquartile range: 6.70–8.90). The value of the third point of the HP standard curve of each plate was plotted in Levey-Jennings charts. Plates that fell outside of the mean +/−2 standard deviations (SD) for two out of three highly immunogenic antigens (GLURP-R2, AMA-1 and MSP-1_19_) were repeated^16^. Logistic regression curves were fitted to standard curve values per plate using the *nplr* package in R Studio^34^, which compares 2 to 5 parameter logistic regression fits and selects the fit with the smallest sum of squared errors. Logistic regression was only fit if no more than one value of the standard curve was missing and at least one of the recorded MFI values was >4.61 (i.e., MFI 100 before log-transformation). MFI values were first converted to proportions using the minimum and maximum MFI values for all standard curves across all antigens (2.07 and 11.17, respectively). The 5-parameter logistic regression is given below:

$$y=B+\frac{T-B}{{[1+{10}^{(b\ast (xmid-x))}]}^{s}}$$

where *B* and *T* are the bottom and top asymptotes, *b* and *xmid* are the Hill slope and the x-coordinate at the inflection point and *s* is an asymmetry coefficient. In 4-parameter logistic regression, the *s* parameter is forced to be 1, while 3- or 2-parameter logistic regression force *B* and *T* to be 0 and 1, respectively. Curve parameters were recorded for each plate as well as a sequence of 200 predicted MFI values across standard curve concentrations to represent the fitted curves.

### Ethics approval and consent to participate

Survey 1 and Survey 3 were approved by the LSHTM Research Ethics Committee (10393), Tulane Institutional Review Board (794709) and the National Bioethics Committee in Haiti (1516-30). The Center for Global Health Associate Director of Science reviewed and approved the protocol for ethical compliance for CDC’s level of engagement. Survey 2 was approved by CDC Institutional Review Board (6821), LSHTM Research Ethics Committee (10466) and the National Bioethics Committee in Haiti (1516-29 and 1617-31). All participants provided informed written consent or assent and blood collection adhered to the approved protocols. All research was performed in accordance with relevant guidelines and regulations.

## Supplementary information

Supplementary information.
