# Supplementary material for: Quality control of multiplex antibody detection in samples from large-scale surveys: the example of malaria in Haiti
Source: Sci Rep. 2020 Jan 24;10:1135. doi: 10.1038/s41598-020-57876-0 (PMC6981173; doi:10.1038/s41598-020-57876-0)
Supplement: Supplementary file 1 — Supplementary information. [file 41598_2020_57876_MOESM1_ESM.pdf]

Supplementary Table

Supplementary Table 1: Frequency of plates with optimal n-parameter logistic regression fit for standard curves per antigen across three surveys in Haiti. Standard curve values were fitted with 2 to 5 parameter logistic regression and the fit with the smallest sum of squared errors was selected. The total number of plates is variable per antigen as logistic regression fits were only applied if no more than one value of the standard curve was missing and at least one of the recorded median fluorescence intensity values was >100 before log-transformation. For antigen abbreviations see Table 1. HP: standard of Haitian hyperimmune sera. NIBSC: WHO *Plasmodium falciparum* 10/198 NIBSC standard.

**Antigen**	**Standard**	**n-parameter**		
		**3**	**4**	**5**
ch150	HP	1	0	354
	NIBSC	0	7	42
dd2	HP	1	4	351
	NIBSC	0	15	35
etr42	HP	0	14	342
	NIBSC	0	6	44
etr51	HP	0	0	357
	NIBSC	0	2	48
gexp	HP	0	6	350
	NIBSC	0	16	34
glurp0	HP	0	1	356
	NIBSC	0	12	38
glurp2	HP	0	10	347
	NIBSC	5	17	28
h103	HP	1	14	198
	NIBSC	0	4	46
hsp	HP	0	10	347
	NIBSC	0	2	48
lama	HP	0	42	315
	NIBSC	0	20	30
lmsp	HP	6	24	326
	NIBSC	2	23	25
lsa	HP	0	0	357
	NIBSC	2	16	32
rcsp	HP	0	4	353
	NIBSC	0	6	44
sbp	HP	0	3	354
	NIBSC	0	6	45
sea	HP	1	13	325
	NIBSC	0	3	47
tt	HP	0	3	354
	NIBSC	2	30	18
pmmsp	HP	0	79	276
	NIBSC	2	12	36
pvmsp	HP	0	8	58
	NIBSC	0	5	23

Supplementary Figures

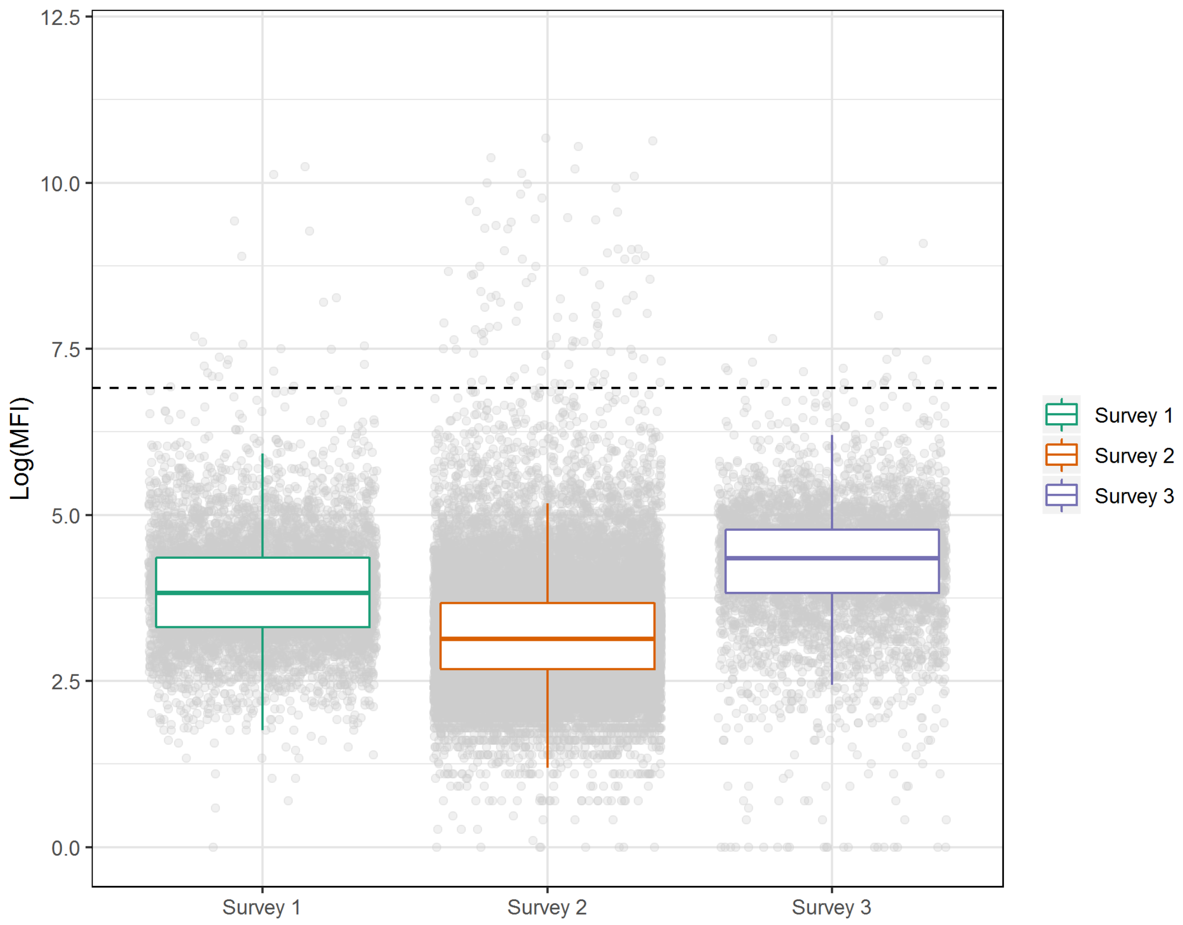

Supplementary Figure 1: Antibody reactivity to the generic glutathione-*S*-transferase (GST) per survey. Median fluorescence intensity (MFI) measurements were corrected for background reactivity of blank responses and natural log transformed. Box plots for GST responses in participant samples are shown per survey, grey dots indicate individual log(MFI) estimates. Participant samples with GST MFI levels above a threshold of 1,000 (dashed horizontal line) were excluded from further analyses as evidence of non-specific binding.

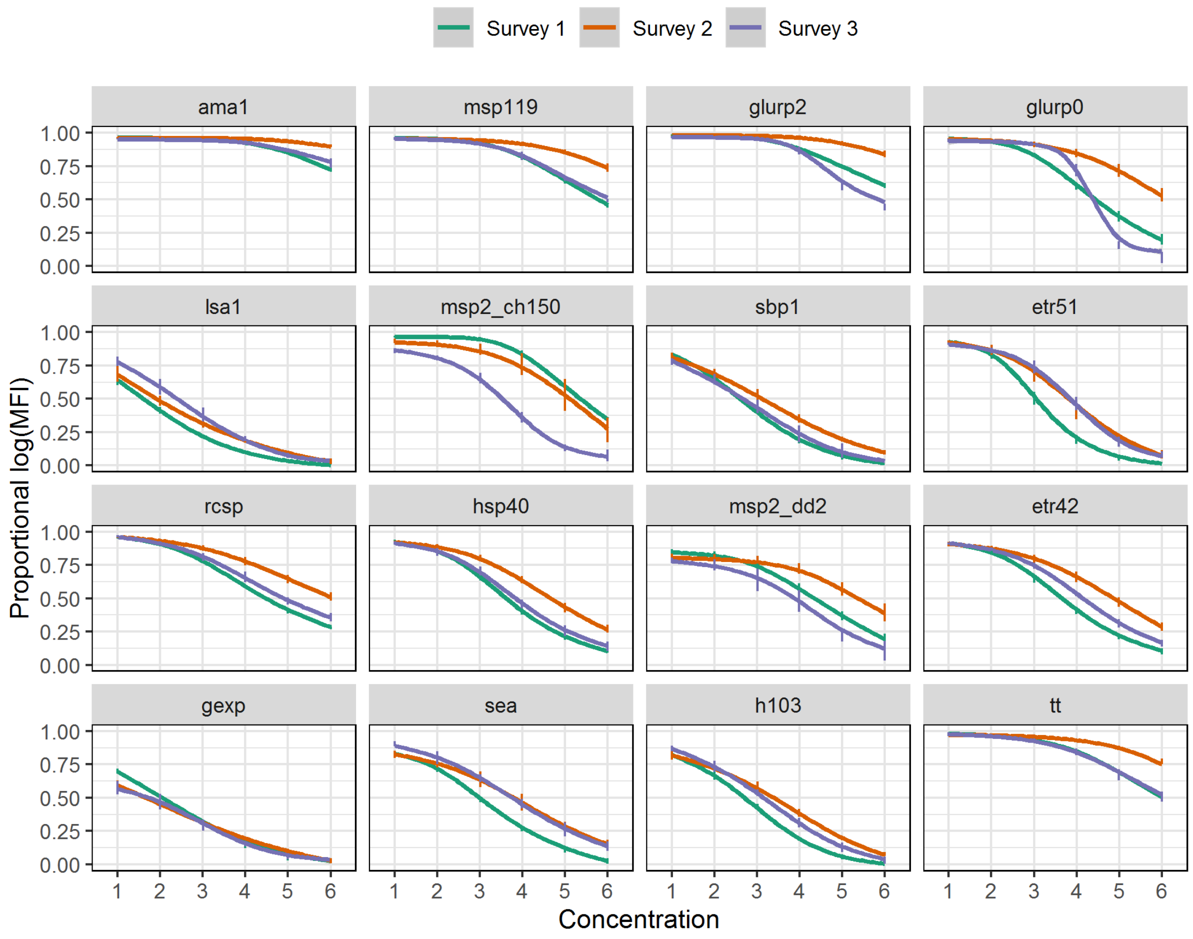

Supplementary Figure 2: Average standard curves of the WHO *Plasmodium falciparum* 10/198 NIBSC standard for each survey. MFI: Median fluorescence intensity; values were corrected for background reactivity of blank responses and natural log transformed. MFI values were converted to proportions using the minimum and maximum MFI values for all standard curves across all antigens (2.07 and 11.17 respectively). For antigen abbreviations see Table 1. For each plate and antigen, NIBSC standard curves were fitted using 5-parameter logistic regression. Standard curves were only fitted if the non-log-transformed MFI of at least one of the dilution points was larger than 100. Using the curve parameters, MFI values were predicted across a sequence of 200 values of standard curve concentrations for each of the plates. Standard curves per survey were plotted using the generalized additive model method and the interquartile range is shown in vertical lines at each of the dilution steps of the standard curve.

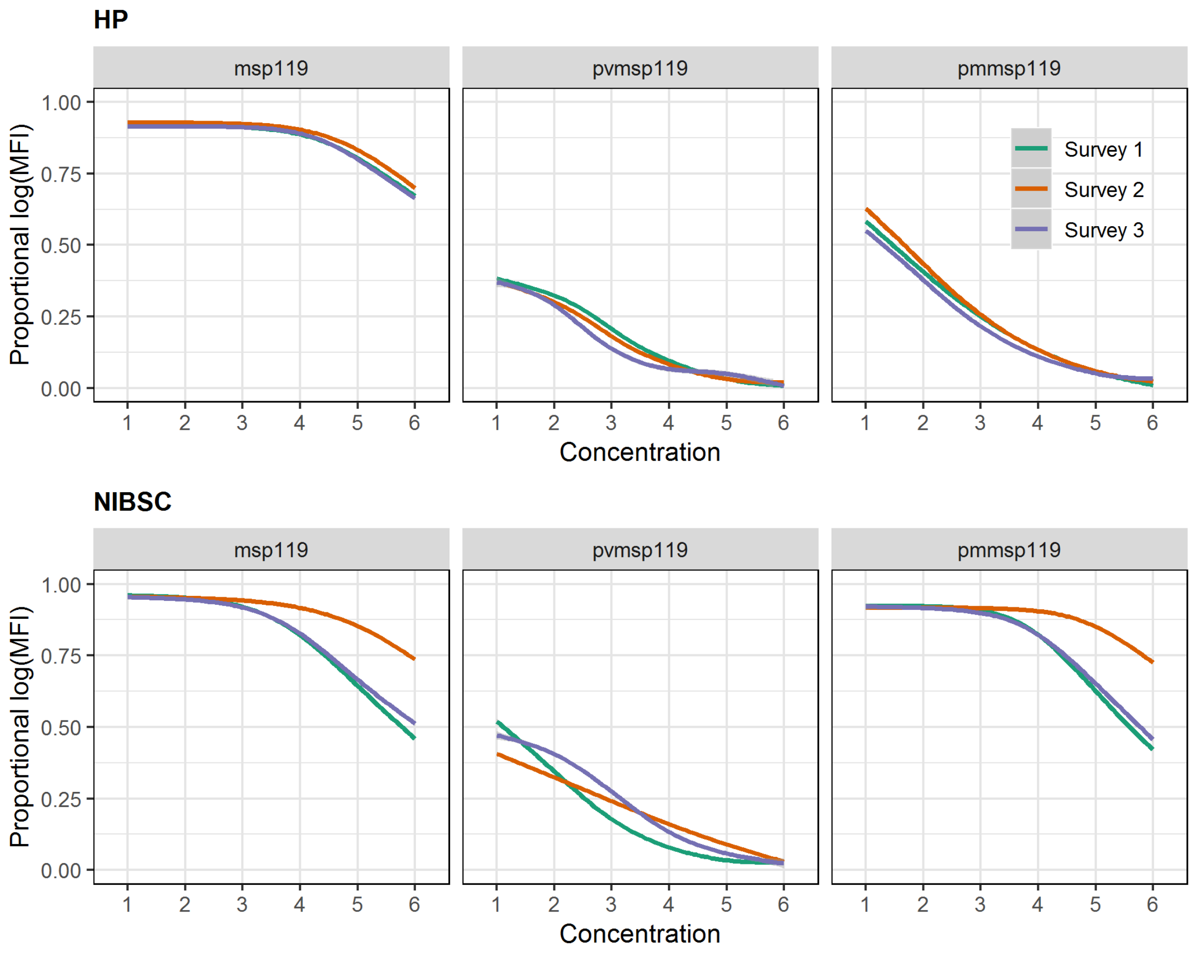

Supplementary Figure 3: Average standard curves of the Haitian hyperimmune sera pool (HP, top) and the WHO *Plasmodium falciparum* 10/198 NIBSC standard (NIBSC, bottom) for each survey for the *Plasmodium falciparum*, *P. vivax* and *P. malariae* 19 kDa merozoite surface protein 1. MFI: Median fluorescence intensity; values were corrected for background reactivity of blank responses and natural log transformed. MFI values were converted to proportions using the minimum and maximum MFI values for all standard curves across all antigens (2.07 and 11.17 respectively). For antigen abbreviations see Table 1. For each plate and antigen, HP and NIBSC standard curves were fitted using 5-parameter logistic regression. Standard curves were only fitted if the non-log-transformed MFI of at least one of the dilution points was larger than 100. Using the curve parameters, MFI values were predicted across a sequence of 200 values of standard curve concentrations for each of the plates. Standard curves per survey were plotted using the generalized additive model method and the interquartile range is shown in vertical lines at each of the dilution steps of the standard curve.
